# Transfer of manualized Short Term Psychodynamic Psychotherapy (STPP) for social anxiety disorder into clinical practice: results from a cluster-randomised controlled trial

**DOI:** 10.1186/s12888-017-1257-7

**Published:** 2017-03-14

**Authors:** Jörg Wiltink, Christian Ruckes, Jürgen Hoyer, Falk Leichsenring, Peter Joraschky, Frank Leweke, Karin Pöhlmann, Manfred E. Beutel

**Affiliations:** 1 Clinic of Psychosomatic Medicine and Psychotherapy, University Medical Center of the Johannes Gutenberg University Mainz, Mainz, Germany; 2 Interdisciplinary Center for Clinical Trials (IZKS), University Medical Center of the Johannes Gutenberg University Mainz, Mainz, Germany; 3 Clinical Psychology and Psychotherapy, Technische Universität Dresden, Dresden, Germany; 4 Department of Psychosomatics and Psychotherapy, University of Giessen, Giessen, Germany; 5 Department of Psychosomatic Medicine and Psychotherapy, Medical Faculty Carl Gustav Carus, Technical University of Dresden, Dresden, Germany

**Keywords:** Social anxiety, Psychodynamic psychotherapy, Manualized, Transfer, Treatment, Cluster-randomised controlled trial

## Abstract

**Background:**

Despite growing evidence for manualized psychodynamic treatments, there is a lack of studies on their transfer to routine practice. This is the first study to examine the effects of an additional training in manualized Short Term Psychodynamic Psychotherapy (STPP) on the outcome in routine psychotherapy for social anxiety disorder (SAD). The study is an extension to a large RCT comparing STPP to Cognitive-Behavioral Therapy of SAD.

**Methods:**

The manualized treatment was designed for a time limited approach with 25 individual sessions of STPP over 6 months. Private practitioners were randomized to training in manualized STPP (mSTPP) vs. treatment as usual without a specific training (tauSTPP). A total of 109 patients were enrolled (105 started treatment; 75 completed at least 20 treatment sessions). Assessments were conducted pre-treatment, after 8 and 15 weeks, after 25 treatment sessions, at the end of treatment, 6 and 12 months after termination of treatment. Remission as primary outcome was defined by the Liebowitz-Social-Anxiety-Scale (LSAS) score ≤30. Secondary outcomes were response (at least 31% reduction in LSAS), treatment duration and number of sessions, changes in social anxiety (LSAS, SPAI), depression (BDI), clinical global impression (CGI), and quality of life (EQ-5D).

**Results:**

Remission rates of mSTPP (9%) resp. tauSTPP (16%) and also response rates of 33% resp. 28% were comparable between the two treatment approaches as well as treatment duration and number of sessions. Most of the within-group differences (baseline to 25 sessions) indicated moderate to large improvements in both treatments; within-group differences from baseline to 12 months follow-up (LSAS, SPAI, BDI, CGI) were large ranging from d = −0.605 to d = −2.937. Benefits of mSTPP were limited to single outcomes.

**Conclusions:**

Findings are discussed with regard to implementation and dissemination of empirically validated treatments in psychodynamic training and practice. SAD patients with a high comorbidity of personality disorders and a long treatment history may need longer treatments.

**Trial registration:**

German Clinical Trials Register (DRKS) DRKS00000570, registered 03. March 2011.

## Background

Social anxiety disorder (SAD) is a highly prevalent disorder with an average 12 month prevalence rate between 2% in the German [1–3] and 7.4% in the US population [4]. Women develop a SAD more frequently than men; mean onset is between 10 and 16.6 years [5, 6]. The course of SAD is usually chronic and full remission is seldom [7]. Affected persons tend to suffer from comorbid disorders such as depression, personality disorders, other anxiety disorders or substance abuse [5]. SAD is often misinterpreted as shyness, unrecognized and undertreated [5, 7].

About half of all psychotherapies in German clinical practice are psychodynamic psychotherapies [8], however, the quality and effectiveness of psychodynamic psychotherapy for SAD is unknown. Many psychodynamic practitioners are biased against structured short-term treatment approaches and disorder specific manualized treatments have rarely been used in psychodynamic training and practice.

In a recent trial of the Social Phobia Network (Sopho-Net funded by the German Federal Ministry of Education and Research, BMBF) remission rates of cognitive therapy, psychodynamic therapy and waiting list were 36, 26 and 8%, respectively. Response rates were 60, 52, and 15% [9]. Cognitive therapy and psychodynamic therapy were equally effective in treating SAD in the long run [10], but there were statistically significant short-term differences in favor of cognitive therapy at the end of treatment in some measures (remission, self-reported social anxiety, interpersonal problems), but not in other measures (response, depression). In addition, the differences were small and below the a priori defined threshold of clinical significance [9].

Dissemination and implementation of efficacious, evidence-based treatments into practice has become a growing issue in mental health care [11]. As psychodynamic manualized treatments have only recently been developed for anxiety disorders, there has been a paucity of research on dissemination and implementation of empirically validated manuals (cf. for an example for panic disorder [12]). It is unknown whether new treatment approaches will improve the effects of routine psychodynamic psychotherapy. However, meta-analytical findings indicate the superiority of strictly manualized STPP [13] compared to mostly non-manualized STPP for the reduction of general psychiatric symptoms [14, 15]. Therefore it is important to investigate how the manualized psychodynamic treatment evaluated in the first funding period of the Social Phobia Network [10] can be transferred from controlled trials into routine clinical care and whether the health care system benefits from such developments.

The central objective of this trial was to analyze the effects of the implementation of manualized Short Term Psychodynamic Psychotherapy (STPP) into routine outpatient care and to test its effects in comparison to common psychodynamic treatment practiced.

We hypothesized that treatment effects reached by private practitioners trained with the manualized procedure of STPP will be superior to therapists who apply their usual psychodynamic treatment and that the implementation of manualized STPP will lead to an average reduction of treatment duration.

## Methods

### Study design

The time period from the start of patient recruitment to completion of follow-up was from April 26, 2011, to June 9, 2015.

In a randomized controlled trial in a naturalistic setting, psychodynamic psychotherapists were randomized either to a training in manualized STPP for social anxiety disorder [16, 17], or to no additional training. Practitioners in the training group (referred to as manualized STPP, mSTPP) received two separate blocks of training, each block with a mean duration of about five clock hours.

The final study protocol and the final version of the written informed consent form were approved by the Ethics Committee of the Statutory Physician Board of Rhineland Palatinate (Germany), which is responsible for the Principal Investigator (Ref. No. 037.249.10 [7258]) and the Data Safety Monitoring Committee (DSMC). The study was monitored by the Interdisciplinary Center for Clinical Trials (IZKS) of the University Medical Center of the Johannes Gutenberg University Mainz. The study was registered at the German Clinical Trials Register (DRKS) DRKS00000570. The study adheres to the CONSORT guidelines.

Detailed information on the study protocol can be found elsewhere [15].

### Recruitment and randomization

#### Study centers

The study was carried out at three trial sites. The participating sites were the Clinic for Psychosomatic Medicine and Psychotherapy of the University Medical Center, Johannes Gutenberg University, Mainz, the Department of Psychosomatics and Psychotherapy, University of Giessen and the Department of Psychosomatic Medicine and Psychotherapy, Medical Faculty Carl Gustav Carus, Technical University of Dresden.

### Therapists

All officially listed (chambers of psychotherapists and medical doctors) psychodynamic psychotherapists in the regions of the trial sites were asked to participate as study therapists. All participating therapists had undergone and completed their training in psychodynamic psychotherapy, which usually takes 5 years to complete. Due to the intended naturalistic character of our study we defined broad inclusion criteria for the participating therapists. Therapists could be of any age and gender. Therapists having participated in the first funding period of the Sopho-Net, and therefore trained and experienced in STPP for social anxiety disorder, were excluded.

A total of 49 therapists participated as study therapists. Twenty-eight of them were female. Therapists reported having practiced as psychotherapists since an average of 14.7 (SD = 10.4) years. Only 12 therapists reported having experience with manualized treatments. There was no significant difference between the therapists treating mSTPP vs. tauSTPP regarding their experience as psychotherapists (M = 14.6, SD 0.6 vs. M = 14.8, SD 11.4 years, *p* = .939) and their number of treated patients during the study (M = 2.3, SD = 1.4 vs. M = 2.1, SD = 1.1, *p* = .588).

All participating therapists were randomly assigned either to a training group which underwent a brief training of the short-term STPP manual for SAD (mSTPP) as developed for the multi-center study or to a control group which continued their routine treatments (non-manualized standard psychodynamic treatment, tauSTPP).

Randomization was stratified taking into account the three trial sites. Randomization of therapists was performed centrally by IZKS Mainz by FAX with a 1:1 randomization ratio using permuted blocks before their training started. Patients were not randomized and did not know whether their therapist was recently trained in STPP for social anxiety disorder or not. As a compensation for their contribution (patient recruitment, ratings), therapists received 200€ per each documented patient.

### Participants

In contrast to other RCTs it was initially planned that patients were recruited by private practitioners and not by the trial sites. For this purpose participating therapists reported patients potentially fulfilling inclusion criteria and willing to participate in the study procedure to the trial site where diagnostics were performed. Due to a delay in recruitment, we decided to additionally recruit through the outpatient clinics of the participating trial sites. In this case, patients first underwent the diagnostic procedure. In contrast to other RCTs participants, they were not randomized to therapists, but they selected their preferred therapist from a list of participating therapists without knowing whether the therapist had been trained in mSTPP or not.

Patients’ inclusion and exclusion criteria are listed in Table 1.

**Table 1:** Inclusion and exclusion criteria

Inclusion criteria	•Diagnosis of SAD (Structured Clinical Interview for DSM, SCID-I, [18]) and Liebowitz Social Anxiety Scale >30 (>60 for generalized subtype) [LSAS, 19, 20] •age: 18 to 70 years •SAD must be primary diagnosis (most severe disorder according to ADIS-IV) •SAD patients with comorbid disorders will be included, provided that SAD is the primary diagnosis, thus ensuring a clinically representative sample as well as analyses of subgroups (e.g. type of SAD, patients with comorbid depressive disorder) •Informed consent
Exclusion criteria	•psychotic disorder •prominent risk of self-harm •acute substance related disorders •personality disorders except for cluster C: avoidant, obsessive-compulsive or dependent personality disorder (SCID-II) •organic mental disorder •severe medical conditions •concurrent psychotherapeutic treatment •psychopharmacological treatment (stable medical treatments; e.g. SSRI without dose adaptation are permitted)

**Fig. 1:**
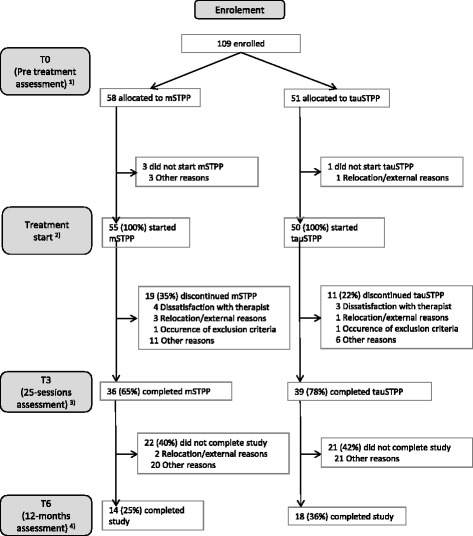
Patient flow. 1) ITT sample 2) mITT sample 3) Finished at least 20 treatment sessions 4) Completer sample

A total of 109 patients were enrolled in the study. One hundred and five patients started treatment and 75 completed at least 20 sessions of the scheduled 25 treatment sessions. A total of 32 patients completed the entire study procedure including a 12 months follow-up. Drop-out rates until 25-sessions assessment were slightly but not significantly higher in the mSTPP group (mSTPP 22/58 vs tauSTPP 12/51, *p* = 0.147, Fisher’s exact test). Fig. 1 shows the patient flow.

There were no significant differences between the two groups regarding gender ratio, age, marital status and education.

Patients treated by therapists trained in mSTPP had more often undergone a psychotherapeutic inpatient treatment in the past and their social anxiety disorder was judged more severe. Table 2 gives an overview of the patient characteristics.

**Table 2:** Patient characteristics (ITT sample, *N* = 109)

Parameter		mSTPP (*N* = 58)	tauSTPP (*N* = 51)	Total (*N* = 109)	*p*-value
Female (%)		29 (50)	24 (47)	53 (49)	.848 +
Age (SD)		33,1 (9.8)	30,7 (8.7)	32 (9.3)	.168 *
Marital status	Single	36 (77)	39 (81)	75 (79)	.746 +
	Married	7 (15)	7 (15)	14 (15)	
	Divorced	4 (9)	2 (4)	6 (6)	
Years at school (SD)		11.9 (1.5)	11.7 (1.7)	11.8 (1.6)	.715 *
Professional education	University degree	12 (20)	14 (27)	26 (23)	.908 +
Severity of primary diagnosis (0–8)	Mean (SD)	6.1 (1.0)	5.6 (1.2)	5.9 (1.1)	.038 *
Number of mental diagnoses	Mean (SD)	1.7 (1.0)	1.4 (1.1)	1.5 (1.0)	.071 *
Personality disorders	N (%)	29 (51.79)	24 (47.06)	53 (49.53)	.625 +
Comorbid diagnoses	Non	5 (8.93)	12 (23.53)	17 (15.89)	.027 +
	One	21 (37.50)	16 (31.37)	37 (34.58)	
	two	16 (28.57)	19 (37.25)	35 (32.71)	
	Three and more	14 (25.00)	4 (7.84)	18 (16.82)	
Treatment history					
Outpatient psychotherapy	N (%)	28 ( 50)	23 (45)	51 (48)	.699 +
Inpatient psychotherapy	N (%)	18 ( 32)	6 ( 12)	24 ( 22)	.019 +
Outpatient psychiatric treatment	N (%)	14 ( 25)	7 ( 14)	21 ( 20)	.150 +
Inpatient psychiatric treatment	N (%)	6 ( 11)	3 ( 6)	9 ( 8)	.491 +

* *p*-values are calculated with the T-Test between treatments according to Satterthwaite

+ *p*-values are calculated with Fisher’s Exact test

### Training

All therapists randomized to mSTPP studied the manual on mSTPP [16, 17]. They were additionally trained in this approach in two workshops of about five clock hours each by AH, FL and PJ. mSTPP therapists were offered site level supervision about every 12 weeks by AH, FL and PJ; tauSTPP therapists were invited to case discussions with the same frequency.

### Interventions

The manualized intervention was based on Luborsky’s Supportive Expressive Therapy (SET) and has been described elsewhere [15–17]. The treatment was customized to 25 individual sessions of STPP over 6 months (short-term therapy, as reimbursed by the German health insurance).

Patients in the control condition received standard psychodynamic treatment.

In both conditions therapists were encouraged to limit treatments to 25 sessions (á 50 min), but due to the naturalistic character of the study their treatments were not limited to 25 sessions. In addition to the aforementioned 25 or more treatment sessions, up to five preparatory sessions (á 50 min) were conducted which are required within the German health care system to cover diagnostic and administrative issues.

### Assessment

Assessments were conducted before treatment started, after 8 and 15 weeks, after 25 treatment sessions, at the end of treatment, 6 and 12 months after termination of treatment. If treatment exceeded 25 sessions an additional post-treatment assessment was performed immediately after termination.

Patients were assessed by independent and trained SCID/LSAS interviewers, who were blind to the treatment condition, i.e. patients did not know, whether their therapists were trained in mSTPP or not. Patients were instructed to not report specific contents of their treatment sessions in order to not reveal the treatment arm to the interviewers. Allocation checks were not performed.

A detailed overview of the assessment in the study can be found elsewhere [15].

### Outcomes

The primary outcome measure was the Liebowitz Social Anxiety Scale observer rating (LSAS-OR [19], German version: [20]). Following recommendations by Liebowitz et al. [21], *remission* was defined by a Liebowitz-Social-Anxiety-Scale score ≤30 [21, 22]. The primary endpoint was the assessment after 25 sessions.

Secondary outcome measures included response to treatment (see below), treatment duration and number of sessions, the LSAS self-rating (LSAS-SR), another scale for the assessment of social anxiety (Social Phobia and Anxiety Inventory, SPAI, [23], German version: [24]) and scales for clinical global impression (CGI, [25]), depression (Beck Depression Inventory, BDI, [26]; German version: [27]), quality of life and social functioning (EQ-5D, [28]).

Response was defined by a 31% reduction (or more) in the Liebowitz-Social-Anxiety-Scale which is comparable to Clinical Global Impression Improvement scale score ≤2 usually used to define response [29]*.*

All instruments were applied in both groups of this trial. Using established cut-off scores for LSAS, the percentages of patients defined as remitted was assessed and statistically compared between the mSTPP and tauSTPP.

### Treatment adherence and treatment differentiation

Due to the naturalistic character of the study we refrained from systematic video- or audiotaping of the treatment sessions; sessions were occasionally videotaped, when therapist and patient both agreed with the recording. In order to determine the therapists’ treatment adherence we devised self-rating scales for patients and therapists based on J. Barber’s Penn Adherence Competence Scale (PACS-SE, [30]). An observer rating of the PACS-SE identifying adherence to supportive, expressive and social anxiety specific interventions has already been applied in the first funding phase of Sopho-Net [9]. Additionally, patients were asked to report activities between treatment sessions. These activities were related to treatment (e.g. I thought about the end of treatment and its relevance for me) or specific interventions of the treatment manual (e.g. I checked my anxiety formula critically). We expected participants treated with mSTPP to score higher on the total scale and on those items referring to interventions from the manual.

The scales were administered after 25 treatment sessions.

### Therapist’s allegiance

Prior to treatment, therapists completed a modified version of the Reaction to Treatment Questionnaire developed by Holt and Heimberg assessing treatment allegiance for every treated patient [31]. Items are scaled from one (not at all confident or logical) to ten (very logical or confident).

### Statistical analyses

At a conservative drop-out rate of 25% (taking into account slightly elevated drop-outs in a practice study), a total of *N* = 105 patients were required to be allocated to the trial. Thus, we initially planned to include *N* = 35 patients in each center. Details on sample size calculation can be found elsewhere [15].

Remission rates were compared between treatments by logistic regression with covariates for treatment, sex, and experience of the therapist. A cluster randomized trial would require an additional term for therapist in the analysis model. However, this was skipped because of the low number of patients per center. The primary analysis was focused on all patients who started the therapy. Missing values were replaced by the last observation carried forward (LOCF) method. Post-hoc the LSAS data analysis was repeated for sensitivity by using multiple imputation. All baseline parameters with a correlation of more than 0.3 were used for imputation of the LSAS values. Remission and response were calculated from the imputed LSAS values. The imputation was repeated ten times by using a chained equation method.

#### Secondary analyses

Self-report questionnaires and observer ratings (SPAI, BDI, CGI and EQ-5D, treatment adherence) were analysed by mixed models with repeated measurements.

All analyses were interpreted on a two-sided level of significance of 0.05.

### Additional analyses

As sensitivity analyses, the analysis was repeated for the per protocol population and a chi-square test was performed. Also the LSAS (observer and self-rating) were analyzed as a continuous measure by means of a mixed model with repeated measurements (MMRM) with fixed effects for treatment, sex and experience of the therapist, week in study and an interaction term of week in study and treatment. This meant especially that data from week 8 and week 15 were included. We used compound symmetry (pre-specified) as a covariance structure and estimated treatment effects within a restricted maximum likelihood (REML). The MMRM allows a fuller use of the data compared to an ANCOVA. Missing values might be predicted by other included data, therefore an MMRM is able to deal with data missing at random (MAR).

As social anxiety was more severe, the mental comorbidity was higher, and pre-treatment inpatient psychotherapy was more frequent in the mSTPP group at baseline, we performed post-hoc subgroup analyses for the above mentioned variables separately by incorporating additional terms for the variable and an interaction term into the mixed model analyses. Between-group differences were not affected significantly by the covariate adjustment of the analyses.

For quantification of effect sizes Cohen’s d for continuous outcomes and Cohen’s h—as a measure of difference between two probabilities or proportions—for dichotomous outcomes within and between treatment conditions were calculated. For the calculation of Cohen’s h the proportion p in each group was transformed by means of γ = 2 arcsin(p). Afterwards the difference between the γ for each group was calculated. The interpretation of the resulting effect sizes is similar to Cohen’s d.

## Results

### Treatment adherence and treatment differentiation

Due to missing data we could analyze a total of 61 adherence questionnaires from the patients’ perspective. For the total scale of 17 items we found a higher agreement to the items (*p* < .001) in mSTPP (M = 14.56, SD = 1.95) compared to M = 11.24 (SD 3.79) in tauSTPP.

As expected behaviours referring to specific interventions of the treatment manual (e.g. I checked my anxiety formula critically) were more often practiced by patients treated with mSTPP. Table 3 gives an overview.

**Table 3:** Adherence/treatment differentiation

	mSTPP (*N* = 27)	tauSTPP (*N* = 34)	*p*-value *
	N (%)	N (%)	
Not specific for mSTPP			
1. I reflected issues from the therapy.	27 (100)	32 (94.12)	.200
2. I practiced what we have discussed in the therapy.	27 (100)	30 (89.24)	.065
7. I thought about demanding high standards of myself.	24 (88.89)	29 (85.29)	.680
8. I thought about situations making me anxious.	27 (100)	28 (82.35)	.022
11. I thought about my behavior in social situations.	27 (100)	32 (94.12)	.200
12. I tried different behavior in such situations.	20 (74.07)	23 (67.65)	.585
13. I thought about my therapist.	21 (77.78)	27 (79.41)	.877
17. I thought about the end of treatment and its relevance for me.	20 (74.07)	21 (61.76)	.309
Specific for mSTPP			
3. I thought about my “anxiety formula”.	23 (85.19)	14 (42.42)	.001
4. I checked my “anxiety formula” critically (e.g. will my fears really become true?)	23 (85.19)	15 (44.12)	.001
5. I thought about encouraging myself.	22 (81.48)	16 (47.06)	.006
6. I practiced encouraging myself.	21 (77.78)	12 (35.29)	.001
9. I went into situations making me anxious.	27 (100)	25 (75.76)	.006
10. I thought about my experiences in these situations.	27 (100)	28 (82.35)	.022
14. I tried not to devaluate myself.	23 (88.46)	26 (76.47)	.234
15. I imagined situations observing me and others on a stage like being among the audience.	10 (37.04)	4 (11.76)	.020
16. I thought about when and why I feel ashamed.	24 (88.89)	20 (58.82)	.009

Analysis Set = mITT Population (*N* = 105)

* *p*-values are calculated with Chi^2^ – Tests

Data of adherence to mSTPP rated by the therapists could be analyzed for a total of 86 patients.

Therapists treating with mSTPP did not differ in their ratings of the frequency of supportive interventions during the treatment (*p* = .415) compared to the tauSTPP therapists (M = 5.63, SD = 0.77 vs. M = 5.50, SD = 0.75).

For the expressive subscale we also could not observe a difference (*p* = .7548) between the treatment settings (mSTPP: M = 5.28, SD = 0.97 vs. tauSTPP: M = 5.22, SD = 0.87).

Both settings differed (*p* < .001) with regards to the frequency of treatment techniques specific for the manualized treatment of social anxiety disorders: M = 5.31, SD = 1.01 for mSTPP vs. M = 4.50, SD = 0.87 for tauSTPP.

### Therapists’ allegiance

Therapists of the mSTPP group reported an average allegiance score of M = 8.61 (SD = 1.11), the tauSTPP therapists of M = 8.17 (SD = 1.38). There was no significant difference (*p* = .108) in the subjective allegiance to treatment.

### Primary outcome

Remission rates (mITT sample) were 9% for mSTPP and 16% for tauSTPP after 25 treatment sessions. Controlled for sex and experience of the therapist in a logistic regression model the difference between the treatment conditions was not significant (OR = .75 [.19–2.93], *p* = .681) with a small effect size of Cohen’s h = −.214. On top of that multiple imputation revealed a non-significant result with *p* = .857.

Remission rates after end of treatment were 13% for mSTPP and 16% for tauSTPP, after 6 months 15% resp. 20%, and after 12 months 22% resp. 26%.

### Secondary outcomes

Response rates (mITT sample) were 33% for mSTPP and 28% for tauSTPP. Again, controlled for sex and experience of the therapist in a logistic regression model the difference between the treatment conditions was not significant (OR = 1.24 [.49–3.16], *p* = .656; Cohen’s h = −.109). On top of that multiple imputation revealed a non-significant result with a notably smaller *p*-value of *p* = .267.

Means and standard deviations for LSAS-OR, LSAS-SR, SPAI, BDI, CGI and EQ-5D at baseline, after 25 sessions and at 12-months follow-up are listed in Table 4.

**Table 4:** Estimated means and standard deviations of scores on social anxiety, depression and psychopathology measures at baseline (T0b), after 25 sessions and 12-months follow-up and effect sizes of between- and within-group differences

Measures			Baseline	25 Sessions	Follow-up	ES (within-group) baseline – 25 sessions	ES (within-group) baseline – follow-up
		Group	M (SD)	M (SD)	M (SD)	d [95% CI]	d [95% CI]
LSAS-OR		mSTPP	77.77 (23.43)	51.85 (23.25	42.87 (27.06)	-.946 [−1.32, −.57]	−1.228 [−1.83, −.62]
		tauSTPP	69.15 (24.71)	55.93 (26.98)	38.18 (23.60)	-.445 [−.84, −.05]	−1.231 [−1.92, −.54]
	ES (between); d [95% CI]		.36 [−.03, .75]	-.16 [−.65, .33]	.19 [−.57, .94]		
LSAS-SR^a^		mSTPP	81.28 (27.70)	54.29 (28.28)	44.57 (27.59)	-.821 [−1.14, −.50]	−1.035 [−1.58, −.49]
		tauSTPP	76.12 (23.94)	52.08 (23.76)	44.81 (22.69)	-.951 [−1.41, −.49]	−1.309 [−2.05, −.56]
	ES (between); d [95% CI]		.20 [−.22, .62]	.08 [−.47, .64]	-.01 [−.78, .76]		
SPAI^b^		mSTPP	5.36 (0.85)	4.38 (1.26)	3.92 (1.12)	−1.023 [−1.35, −0.69]	−1.211 [−1.85, −.57]
		tauSTPP	4.94 (0.93)	4.22 (1.16)	3.67 (1.36)	-.668 [−1.01, −0.32]	−1.141 [−1.77, −.51]
	ES (between); d [95% CI]		.47 [.04, .89]	.14 [−.38, .65]	.19 [−.54, .92]		
BDI^c^		mSTPP	18.05 (8.14)	10.65 (7.08)	7.41 (6.38)	-.704 [−1.12, −.29]	−0.985 [−1.62, −.35]
		tauSTPP	18.50 (10.29)	13.44 (9.44)	11.81 (13.03)	-.564 [−0.87, −.26]	−0.605 [−1.04, −.17]
	ES (between); d [95% CI]		-.05 [−.47, .37]	-.33 [−.84, .18]	-.43 [−1.18, .32]		
CGI^d^		mSTPP	5.22 (0.73)	3.87 (1.02)	3.21 (1.31)	−1.908 [−2.57, −1.24]	−2.937 [−4.20, −1.67]
		tauSTPP	4.80 (0.78)	3.63 (1.31)	3.08 (1.44)	−1.498 [−2.13, −.86]	−2.193 [−3.29, −1.10]
	ES (between); d [95% CI]		.55 [.15, .95]	.21 [−.32, .73]	.10 [−.69, .89]		
EQ-5D^e^		mSTPP	48.47 (32.81)	55.00 (36.36)	77.27 (15.42)	.073 [−.33, .47]	.782 [−.08, 1.64]
		tauSTPP	59.68 (26.39)	61.90 (31.82)	49.14 (38.47)	.142 [−.22, .51]	-.719 [−1.34, −.10]
	ES (between); d [95% CI]		-.38 [−.77, .02]	-.20 [−.67, .27]	.90 [.20, 1.61]		

A posteriori power for the difference after 25 sessions and baseline (α = 5% one-sided, two-sample t-test): ^a^ 17%, ^b^ 55% ^c^ 42% ^d^ 55% ^e^ 24%

Analysis Set = mITT Population (*N* = 105)

Most of the within-group differences (baseline to 25 sessions) were large for both treatment settings (mSTPP and tauSTPP). Only LSAS-OR for tauSTPP showed a decline with a small effect size of d = −.445 from baseline to 25 sessions, and EQ-5D showed only a small increase for both treatment groups.

Most of the effect sizes of the long-term within-group differences (baseline to 12 months follow-up) were larger than the short-term within-group differences (baseline to 25 sessions) ranging from d = −0.605 to d = −2.937.

EQ-5D showed a large improvement in mSTPP and a large decrease in tauSTPP from baseline to follow-up. Table 4 gives an overview.

All between-group differences after 25 treatment sessions were small to medium with effect sizes ranging between −0.33 and 0.21 (see Table 4).

For the between groups differences at 12-month follow-up we found small effects for LSAS-OR, LSAS-SR, SPAI, CGI, and BDI (see Table 4).

Quality of life (EQ-5D) was significantly higher in patients treated with mSTPP 12 months after treatment (M = 77.27, SD = 15.42 vs. M = 49.14, SD = 38.47 for tauSTPP). The effect size for this difference was large (d = 0.90). Table 4 gives an overview.

### Treatment duration

The mean duration of treatments was comparable (*p* = .602): 68.13 weeks (SD 41.23) in mSTPP and 63.56 weeks (SD = 28.34) in tauSTPP. The time until 25 sessions did not differ between the two groups (*p* = .373): 37.68 weeks (SD 14.70) in mSTPP and 40.58 weeks (SD = 12.90) in tauSTPP. A total of 29 (35.4%) treatments exceeded 25 sessions.

The total number of sessions until treatment ended did not differ significantly between the treatments: 36.35 sessions (SD = 34.48) for mSTPP and 33.86 sessions (SD = 23.52) for tauSTPP (*p* = .7047).

## Discussion

As the first of this kind, our study compared the effects of manualized STPP and STPP as usually being practiced by therapists in their private practice in a sample of patients with SAD.

Allegiance to treatments was high and did not differ significantly between the two groups.

Due to the naturalistic character of our study we did not perform regular adherence checks to ensure treatment integrity. However, from the patient’s perspective we could differentiate treatments by retrospective assessment of their activities between the therapy sessions. Furthermore, therapists reported a higher adherence to interventions specific for the treatment of social anxiety in the mSTPP group.

We hypothesised that the manualized procedure of STPP will be superior to usual psychodynamic treatment and lead to an average reduction of treatment duration. In our naturalistic setting remission rates after 25 treatment sessions (mSTPP 9%, tauSTPP 16%) did not differ significantly between the two treatment groups. Treatments were not shorter compared to tauSTPP. Effect size differences between the two treatment groups were small for most secondary outcomes. However, most effect sizes were—albeit insignificantly—higher in the mSTPP group from baseline to treatment termination. Depression was lower and quality of life was higher after a follow-up of 1 year in the mSTPP group. Indeed, quality of life had moderately increased in mSTPP and moderately decreased in tauSTPP.

These results can be seen as being disappointing. While moderate to large effect sizes were achieved for most outcomes in both conditions, the rates of response and remission were low and below the findings from the main study [9].

The transfer of new therapeutic strategies from an RCT was neither superior regarding the main outcome nor regarding the duration of treatment. Reasons for this lack of differences are multifarious. Firstly, despite of randomization of therapists, patients in the mSTPP group were more seriously ill regarding the severity of the social anxiety disorder and also had more comorbid diagnoses, which was likely to adversely affect remission rates [32, 33] and also may have caused the slightly, but not significantly higher drop-out rate in the mSTPP group. Secondly, compared to the study of Leichsenring et al. [9], in this study, therapists did not treat pilot patients with the manual before starting with study treatments, and frequency of supervision was much lower. Therefore, we assume that the familiarity with the manual was also lower in this study. Thirdly, we did not restrict treatments to 25 sessions. As usual in German routine care, therapists were free to decide about treatment duration based on their professional evaluation. Maybe, therapists have acted less straight forwardly in conducting their therapy, because they were free to extend the therapy.

In the previous randomized controlled trial [9] we found higher remission rates (26%) for mSTPP after 25 treatment sessions, almost comparable to our current remission rates after 12 months. However, baseline to 25 sessions effects for the improvement of social anxiety (LSAS-OR, LSAS-SR and SPAI) were almost high ranging between -.445 and −1.023) with an increase to 12 months follow-up (baseline to follow-up) for both treatments. The large effects in secondary outcomes and the corresponding low remission rates indicate a lower variance of social anxiety in our study compared to the study of Leichsenring et al. [9]. One reason for the lower variance in this study may be due to the fact that the participants in this study showed more severe presentations of SAD. Compared to the participants treated with mSTPP in the study of Leichsenring et al. [9] participants in our study suffered from higher depression scores (BDI), a 2-fold higher rate of personality disorders and more comorbid mental disorders (for comparison see [32]). For such a highly distressed population reaching the cut-off of 30 points on the LSAS is much more difficult.

Another reason for the lower remission rates after 25 treatment sessions (resp. delayed increase in remission rates during follow-up) might be associated with the broader inclusion criteria (age up to 70 years, stable medical treatments, e.g. SSRI without dose adaptation were permitted) in our study, also leading to a more difficult to treat population.

Short-term (25 sessions) and long-term (12 months) effects on depression (BDI) and especially clinical global impression (CGI) are encouraging. Both conditions led to further improvements following treatment termination. However, the manual mainly focused on symptoms of social anxiety disorder. There were no short term improvements of quality of life (QoL) in both treatments. The increase of QoL in the mSTPP and the decrease in the tauSTPP group is the only difference between the settings and should therefore be interpreted with caution because of the substantial data loss. Given the slightly higher effect sizes for most of the outcome criteria for mSTPP during treatment, it could be surmised that improvement of QoL also took place earlier compared to tauSTPP.

The strength of the study is its naturalistic character; being much closer to real life conditions of therapists in their private practice than under strongly controlled RCTs. We would presume that this also led to the inclusion of more severely impaired patients compared to the main RCT. In order to ensure feasibility of the trial in terms of dissemination and implementation, we purposefully limited the effort for the participating therapist (no training case, shorter training, fewer supervision) compared to the main study. Thus, there are several limitations. Firstly, due to the naturalistic character we also did not do stringent adherence checks, which humbles the internal validity of the study (differences between the treatment arms are somewhat vague), but also increases its generalizability describing more realistic conditions of private practice. Secondly, only 49 therapists participated in the study, which may cause a selection bias by motivated therapist or those interested in research activities. Thirdly, a relatively high drop-out rate and the necessary change of the recruitment strategy due to delay in recruitment might decrease the generalizability of the results.

Despite its limitations this study is a promising attempt to study the transfer of manualized treatments into clinical practice.

We may conclude that the implementation of manualized treatments pursued was not sufficient to reach treatment effects of strictly controlled randomized controlled trials. This raises the issue how dissemination and implementation could be improved. Our strategy was to keep demands on therapists low; we cannot be sure if raising the demands of training for treatments for specific disorders (as in our previous RCT) would really be acceptable for the majority of therapists in private practice. As alternatives, one might consider developing transdiagnostic manuals suitable for a range of disorders [34], devising more comprehensive self-learning materials (e.g. complementing written manuals by videotapes of treatments) and—perhaps most importantly—introducing manualized treatments into psychodynamic training and thereby a) increasing the openness of using specific manuals, b) increasing the willingness to work in a time-limited and focused treatment framework and c) ensuring that the duration for training on manualized approaches exceeds the 10 h of our study and therefore hopefully ensures a more competent delivery of the treatment.

Our results might suggest that alternative treatment strategies should be preferred for patients with a higher symptom severity. One strategy could be a longer treatment duration for a better outcome. It is an intriguing question for future studies on the efficacy of PDT in social anxiety disorder, whether a symptom severity adapted treatment duration increases treatment effects. As recent guidelines [35] or meta-analyses (e.g. [36]) suggest, a combination of psychotherapy and medication treatment may also be helpful for those patients with anxiety disorders with worse outcome or higher initial symptom severity.

## Conclusions

The findings of our study indicate that the necessary effort for the successful implementation of manualized treatments is high. More comprehensive self-learning materials and the introduction of manualized treatments into psychodynamic training might be promising attempts.

Alternative treatment strategies should be preferred for those patients with a higher initial symptom severity and/or comorbid personality disorders. Possible treatment strategies could be longer treatment duration or combination of treatments (e.g. psychotherapy and medication).
